# Effect of Waveguide Aperture and Distance on Microwave Treatment Performance in Rock Excavation

**DOI:** 10.3390/s23041929

**Published:** 2023-02-09

**Authors:** Fangfang Chen, Zhanqiang Wu, Zhiqiang Zhang

**Affiliations:** 1 Xi’an Key Laboratory of Geotechnical and Underground Engineering, Xi’an University of Science and Technology, Xi’an 710054, China; 2 School of Architecture and Civil Engineering, Xi’an University of Science and Technology, Xi’an 710054, China; 3 School of Civil Engineering and Architecture, Xi’an University of Technology, Xi’an 710048, China

**Keywords:** microwave-assisted rock breaking, waveguide aperture, irradiation distance, waveguide aperture, rock temperature, rock damage

## Abstract

Rock burst is a common hazard during tunnel excavation in high-stress and hard rock strata. Microwave-assisted breaking has a great potential application in hard rock tunnel excavation, reducing the possibility of rock burst, and how to reasonably make the application on the TBM cutterhead is one of the critical issues. The waveguide aperture and distance between the rock face and waveguide have serious effects on its performance. In this paper, based on the arrangement of the microwave waveguide of the TBM cutterhead and the actual excavation situation, considering the reflection of microwave energy by the metal cutterhead and the scattering state of electromagnetic waves at the rock surface irradiation, a 2D model of rock irradiated by microwaves is established. The effects of waveguide aperture and distance on microwave irradiation performance of rock are studied, considering three different waveguide types: convergent waveguide, rectangular waveguide, and horn waveguide. The results show that the maximum temperature is located on the rock irradiation surface, rather than inside the rock. The rock temperature decreases in a cosine pattern with irradiation distance, rather than in linearity, which is consistent with the characteristics of electromagnetic wave propagation. The interval of irradiation distance where the rock temperature local maximum value appears is 1/4 of the electromagnetic wavelength, corresponding to the crest and trough of the electromagnetic wave. The rock temperature at the wave trough distance is lower than that of the wave crest distance, but the high-temperature zone is wider. In the range of 50~200 mm waveguide apertures, the rock temperature and damage decrease with the increase in waveguide aperture when irradiated at the crest distance, while the valley distance is opposite. A convergent waveguide and short irradiation distance enhance the energy focusing performance, so the temperature rise characteristics and rock damage are more concentrated. A large-waveguide-aperture horn waveguide and long irradiation distance form a wide range of high-temperature zones and rock damages.

## 1. Introduction

Microwave heating technology is an efficient and green heating treatment technology. Using microwave energy to weaken rocks has become an important research topic in the last 50 years, and it is widely considered as an auxiliary excavation technology with sustainable development and promising utilization prospects in the future [1]. In the process of underground engineering excavation, rock burst triggered by high ground stress causes casualties and equipment damage. The mechanical tool and cutterhead cannot break hard rock with ease, which seriously increases the construction difficulty and cost. During the excavation of the Qinling Water Conveyance Tunnel of the Han to Wei River Diversion Project, which has attracted much attention in China, the hard rock encountered in tunnel engineering was mostly metamorphic rock dominated by granite and basalt. In this type of situation, the cutterhead is seriously worn out, the tunnel boring machine (TBM) is damaged, and other problems sometimes occur. According to statistics, the cutter cost accounts for 1/3 of the tunnel construction cost [2], and the excavation advance rate is reduced to 0.36 m/h at least. The rock burst disaster and rock wall failure of the intake tunnel of NJHEP Hydropower Station in Pakistan also increased the difficulty of TBM excavation [3]. Microwave heating can weaken rocks, improve mechanical excavation efficiency, generate fractures or cracks in the rock, release high ground stress [4], and significantly reduce the excavation difficulty and cost [5]. Some field tests proved that microwave-assisted rock breaking research also had a certain feasibility. Gushchin et al. [6] designed a combined boring machine that integrates the advantages of microwave and mechanical construction. The field mining test of a phosphorus ash mine concluded that the microwave-assisted tunneling speed was significantly improved compared with the single tunneling method. Protasov et al. [7] also carried out tunnel driving tests by using microwave- and mechanical-combined boring machines and found that the speed of combined tunneling was 1.5 times higher than that of mechanical tunneling. Lu et al. [8] studied the effect of microwave irradiation on rock breaking of the TBM cutterhead and proved that microwave irradiation can reduce the energy consumption of the rock breaking effect of the TBM, and the application of the microwave-radiation-assisted TBM to cut hard rock was feasible both in theory and practice. In addition, intergranular cracks are generated at the mineral boundary by microwave heating, which is conducive to effective mineral separation and recycling [9]. Microwaves can also be used to recycle concrete coarse aggregate [10] or dismantle concrete structures [11], and have excellent potential application in the civil industry and other engineering fields.

Many scholars have made considerable contributions to the study of the factors and trends that influence the effect of microwave irradiation on rocks, which provides important references for how to improve the effect of microwave-assisted rock breaking and optimize this technology. According to Lu [12] et al.’s experiment of irradiating basalt in a microwave cavity, the sample’s temperature increases obviously with the increase in microwave energy input, and the number and propagation speed of cracks obviously increase as well. Toifl et al. [13] analyzed the influence of microwave irradiation parameters on the temperature and stress of granite by numerical simulation, and also observed that, if the input energy is constant, the temperature and stress caused by high-power irradiation are larger. Dai et al. [14] conducted considerable studies to obtain similar results. In addition, it was also found that, if the rock sample is in a saturated state, the sample damage is more serious [15].

**Figure 1:**
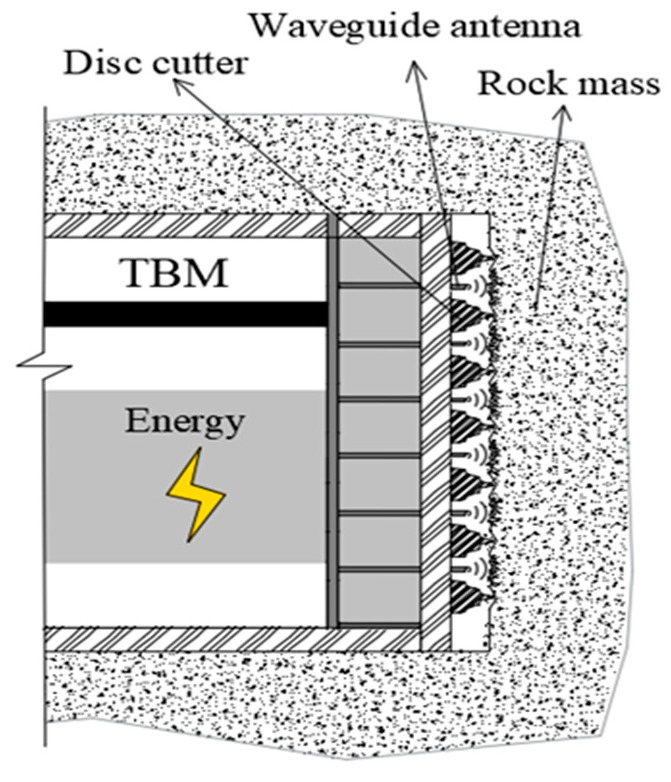
Schematic diagram of “microwave and mechanical” excavation.

However, most of the experimental studies on rock irradiated by microwaves are carried out mostly in cavities; the rock sample is placed in a microwave cavity, which is inconsistent with the scene of rock excavation by the microwave-assisted cutterhead, as shown in Figure 1. Unlike the experiment of rocks irradiated by a microwave cavity, the propagation and reflection characteristics of electromagnetic waves irradiated by an open surface are different, and the heat transfer effect of the rock sample is also different, so the temperature rise characteristics and damage characteristics are greatly different. In the workplace, the research on microwave open-surface irradiation has better engineering significance. Therefore, it is necessary to focus on how to reasonably arrange the waveguide on the cutterhead to improve the weakening effect, and explore the rules and mechanisms of different arrangement factors, such as irradiation distance and waveguide aperture, on rock irradiation.

Liu [16] established a z-D model of a rectangular waveguide irradiating rock based on the TBM cutterhead, and observed that the irradiation distance can cause an electromagnetic wave energy distribution on rock sample surface, forming different peak points, and the sample plastic zone was also closely related to peak points. Grafe et al. [17] found that a rectangular waveguide usually needs a high power level to break granite, while a horn waveguide has the advantage of radiating microwave intensity and irradiating a larger area of rock. Lu et al. [18] used the self-made convergent waveguide to illuminate basalt samples in an open way and found that the device had the function of energy gathering, which significantly improved the heating rate and crushing effect of the samples. Ma et al. [19] confirmed this phenomenon through numerical simulation experiments of a rock sample irradiated by a convergent waveguide with different heights, and with the increase in irradiation distance, the high-temperature area and the highest temperature of the sample decreased rapidly. In addition, it was found that the smaller the convergent waveguide height, the higher the sample surface temperature, but the smaller the high-temperature range. Zhang [20] studied the design and performance of a convergent waveguide, proving that the energy density produced by the convergent waveguide is high, but the range is small. Hassani et al. [21] used a horn waveguide to irradiate stacked basalt slabs at different distances through a microwave cavity irradiation experiment. It was found that the surface temperature of the samples decreased linearly with the increase in irradiation distance, but the reflected energy decreased in sinusoidal form and the diameter of the irradiated area corresponded to the waveguide aperture of the horn waveguide. Pressacco et al. [22] studied the 2D simulation of the horn waveguide irradiating rock at different positions. When the horn waveguide is used to irradiate the rock sample, a considerable crack failure can be generated under the conditions of longer heating time or higher microwave power.

**Figure 2:**
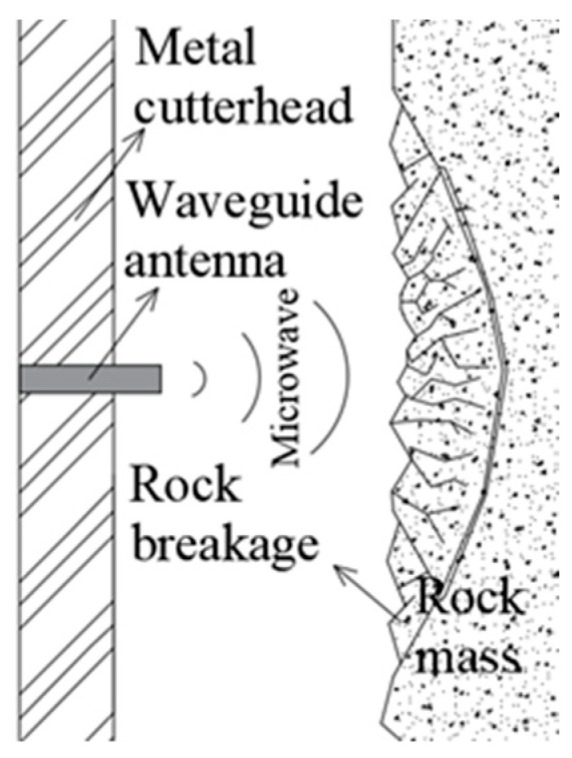
Schematic diagram of microwave open-ended irradiation on rock mass.

To sum up, different waveguide types have their own advantages and disadvantages on the weakening effect of rock, which needs comprehensive comparative evaluation, and the evaluation of irradiation distance also needs to be combined with the actual excavation work. The temperature rise of rocks may be better in short-range irradiation, but the high-temperature radiation generated by short-range irradiation on the cutter head will also affect the normal operation of the tool equipment and construction safety. The existing research also neglects the influence of the interaction between waveguide apertures and irradiation distances on the rock irradiation effect, and the comprehensive evaluation and analysis of waveguide form on the rock weakening effect. In this paper, based on the actual scene of open microwave irradiation on rock (as shown in Figure 2), considering the reflection effect of the cutterhead on electromagnetic waves, the differences in irradiation effect caused by the form of the waveguide and its aperture are studied, especially in the electric field distribution, high-temperature zone, and plastic zone. The influence of microwave irradiation distance is also considered.

## 2. Materials and Methods

### 2.1. Modeling

**Figure 3:**
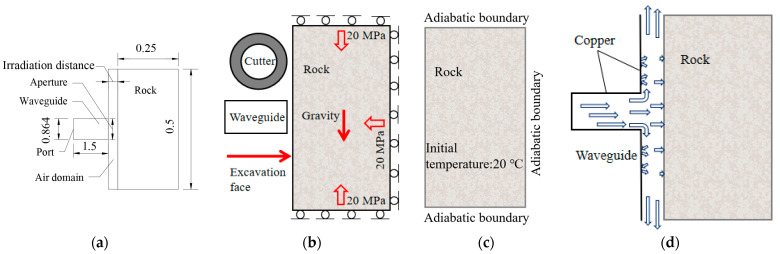
Schematic diagram of model geometry and boundary conditions. (**a**) Main parts and size (m); (**b**) mechanical BCs; (**c**) thermal BCs; (**d**) electromagnetic BCs.

COMSOL was applied to simulate the 2D microwave irradiation on the rock in this paper. Based on the coupling of electromagnetics, thermodynamics, and mechanics, the influences of different waveguide apertures and irradiation distances on the temperature, electric, and damage field distribution of the rock were analyzed. The geometric model simplified according to the TBM cutterhead is shown in Figure 2, which consists of the waveguide, air domain, and rock, as shown in Figure 3a.

In this paper, the microwave frequency was 2.45 GHz, the wavelength was 12.2 cm, and the microwave port was WR340 model. The mode was TE10 and the width was 86.4 mm. The length of the waveguide was 150 mm. The rock sample was assumed to be intact, homogeneous, isotropic, and dry. The rock sample was rectangular in shape with a height × width of 0.5 m × 0.25 m, respectively, and the width of the air domain represents irradiation distance, as shown in Figure 3a.

The left boundary of the rock sample was the excavation surface and set to be free; the upper, lower, and right boundaries were provided with roller supports, as shown in Figure 3b. To meet the initial stress state, a 20 MPa prestress was added on the rock and gravity was set. The initial temperatures of the rock sample and air domain were set to 20 °C. The non-irradiation boundary and the upper, lower, and right boundaries of the rock sample were set to be adiabatic, as shown in Figure 3c.

The left boundary of the air domain was the metal cutterhead; the waveguide boundary and the left boundary of the air domain were made of copper; the impedance conditions were set to simulate the reflection of electromagnetic waves. The upper and lower boundaries of the air domain were non-impedance, set as the scattering conditions of divergent electromagnetic waves and matching the real excavation condition. The propagation path of the model electromagnetic energy was, thus, obtained, as shown in Figure 3d. The arrow direction in the figure shows the propagation path of the electromagnetic wave.

The models of the rock sample irradiated by different waveguide apertures and distances were established. The irradiation times were 60 s and 120 s, and the power was 100 kW. Generally, the diameter of the hob was 300~483 mm, so the irradiation distance of the microwave waveguide within 25 cm was appropriate. In order to explore the influence of irradiation distance on the effect of microwave irradiation on the rock, the irradiation effect of a rectangular waveguide at the irradiation distance of 25~50 cm was also simulated. In order to explore the change in rock temperature rise and plastic zone with the aperture of the waveguide, the aperture range of the microwave waveguide was considered to be the same as the height of rock, so the aperture range of the waveguide was 10~500 mm, of which 10~86.4 mm was the convergent waveguide, 86.4 mm was the rectangular waveguide, and 86.4~500 mm was the horn waveguide. The physical and mechanical parameters of the rock sample are listed in Table 1.

### 2.2. Governing Equations

Minerals with high dielectric properties in rock absorb electromagnetic wave energy and convert it into heat energy [23]. As the temperature of rock rises, thermal expansion is restricted by the surrounding rock to cause thermal stress, and the rock is damaged when the stress reaches the yield strength. In COMSOL, the numerical calculation of the microwave irradiation on the rock was achieved through the coupling of Electromagnetics-Thermology-Mechanics (E-T-M), and the electromagnetic field propagates through Maxwell equations:

(1) $$\nabla \times \mu_{r}^{-1}\left(\nabla \times E\right)-k_{0}^{2}\left(\varepsilon_{r}-\frac{j\sigma }{\omega \varepsilon_{0}}\right)E=0$$

where ∇× is the curl operator; E is the electric field strength; V/m, $\varepsilon_{0}$ is the vacuum dielectric constant, 8.85 × 10^−12^ F/m; $\mu_{r}$ is the relative permeability; $k_{0}=\omega /c_{0}$ is the vacuum wave number; $c_{0}$ is the light speed in vacuum, 2.998 × 10^8^ m/s; $\varepsilon_{r}$ is the relative dielectric constant; σ is the electrical conductivity, S/m; ω is the angular frequency, rad/s. In the process of microwave propagation, the microwave energy P absorbed by the rock per unit volume can be expressed as:

(2) $$P=\pi f\varepsilon_{0}\varepsilon^{\prime \prime }E^{2}$$

where f is the microwave frequency. The dielectric properties of the dielectric are described by the complex relative dielectric constant $\varepsilon_{r}=\varepsilon^{\prime }-j\varepsilon^{\prime \prime }$, where $\varepsilon^{\prime \prime }$ is the imaginary part of the dielectric constant and $\varepsilon^{\prime }$ is the real part of the dielectric constant. The ratio of the imaginary part to the real part of the dielectric constant represents the ability of the dielectric to lose microwave energy. Heat conduction after microwave irradiation is described by the Fourier energy balance equation:

(3) $$\rho C_{P}\frac{\partial T}{\partial t}-k\nabla^{2}T=P$$

where ρ is the density, kg/m^3^; $C_{P}$ is the specific heat capacity, J·(kg·K); T is the temperature, K; k is the thermal conductivity, W/(m∙K). The microwave energy absorbed by the medium is converted into heat energy, and the temperature rise can be expressed as:

(4) $$\frac{\Delta T}{\Delta t}=\frac{P}{\rho C_{P}}$$

The strain η caused by thermal expansion due to the change in temperature is:

(5) η=αΔT

where α is the thermal expansion coefficient, 1/K. The thermal stress τ caused by thermal expansion is calculated using Hooke’s law:

(6) $$\tau =\frac{\eta E}{1-2\mu }$$

where E is the elastic modulus, GPa; μ is Poisson’s ratio. The penetration depth Z of microwaves in the medium is related to the wavelength and dielectric properties:

(7) $$Z=\frac{\lambda_{0}\sqrt{\varepsilon^{\prime }}}{2\pi \varepsilon^{\prime \prime }}$$

where $\lambda_{0}$ is the electromagnetic wave wavelength. The penetration depth of microwaves in the rock medium is generally about 5 cm. Through the penetration and heat transfer of microwaves in the rock, the rock is heated and expanded to produce stress, which is destroyed after reaching the limit equilibrium. Assuming that the rock is an elastic–plastic material, the Mohr–Coulomb strength criterion is adopted to determine the plastic failure under microwave irradiation:

(8) $$\frac{\sigma_{1}-\sigma_{3}}{2}=\left(c\cdot ctg\varphi +\frac{\sigma_{1}+\sigma_{3}}{2}\right)\sin\varphi$$

where $\sigma_{1}$ and $\sigma_{3}$ are the principal stresses; c is the cohesion, MPa; φ is the internal friction angle, rad.

## 3. Results

### 3.1. Electromagnetic Field

The electromagnetic wave propagation is one of the fundamental factors that affect the temperature and damage in a rock sample. The electromagnetic wave is reflected continuously in the waveguide, and the convergent waveguide concentrates the electromagnetic wave energy into a certain range at the waveguide port. The horn waveguide diverges the electromagnetic wave, and the wave peaks and troughs appear in the longitudinal propagation process with the increase in the aperture. Rectangular waveguides transmit uniform electromagnetic waves. The vertical propagation process of electromagnetic waves changes with different waveguide apertures, and the horizontal propagation process also changes due to different irradiation distances.

**Figure 4:**
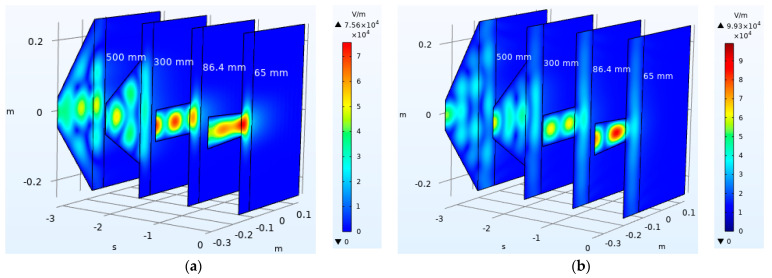
Electric field distribution in the rock sample and waveguide aperture. (**a**) Irradiation distance of 5 cm; (**b**) irradiation distance of 8 cm.

Based on Equations (1)–(8), the rock temperature distribution, model electric field distribution, and plastic zone distribution of the rock under microwave irradiation were obtained by the coupling solution set with the appropriate boundary and initial conditions. By comparing the crest distance of 5 cm and the trough distance of 8 cm, the electric field distribution of the model is shown in Figure 4 under the irradiation of the waveguide apertures of 65 mm, 86.4 mm, 300 mm, and 500 mm. It can be seen that the electromagnetic wave propagates in a rectangular waveguide with a stable cosine trend. However, the convergent waveguide and the horn waveguide interfere with this state. After reaching the waveguide port, the electromagnetic wave has different divergence characteristics due to the different irradiation distances.

At the irradiation distance of 5 cm, the energy of the electromagnetic wave concentrates on the local surface of the rock sample, resulting in a large temperature rise and damage of the rock sample. The increase in waveguide aperture can increase the surface area covered by electromagnetic waves but reduces the electromagnetic wave energy. When the radiation distance is 8 cm, the electromagnetic wave energy is propagated from the waveguide opening to the entire sample surface. However, the overall electromagnetic wave energy is weak, and the sample temperature and damage are lower.

As the rock sample is at the crest distance of the electromagnetic wave at the irradiation distance of 5 cm, the maximum value of the electromagnetic wave is in the air domain, which causes the electromagnetic wave energy to diverge in the air domain and the peak value to decrease. However, under the irradiation of 8 cm trough distance, the maximum value of the electromagnetic wave is in the waveguide, resulting in a relatively concentrated energy and high peak value. Therefore, the electric field peak value of the 65 mm waveguide aperture is 7.56 × 10^4^ V/m at an irradiation distance of 5 cm, while the electric field peak value is 9.93 × 10^4^ V/m at an irradiation distance 8 cm. This is closely related to the irradiation distance because the electric field distribution is mainly related to the dielectric properties of the medium; to be precise, it is the real part of the rock’s dielectric constant. Although the permittivity of the rock is unchanged, the change in the radiation distance indirectly leads to the position of the rock in the model, which affects the spatial distribution state of the electric field. The increase in waveguide aperture results in the multi-point distribution (5 cm) or global coverage (8 cm) of electromagnetic wave energy on the rock surface, which makes the rock sample generate a larger high-temperature zone through heat transfer and form a larger damage area.

### 3.2. Rock Sample Temperature Irradiated by Different Irradiation Distances

**Figure 5:**
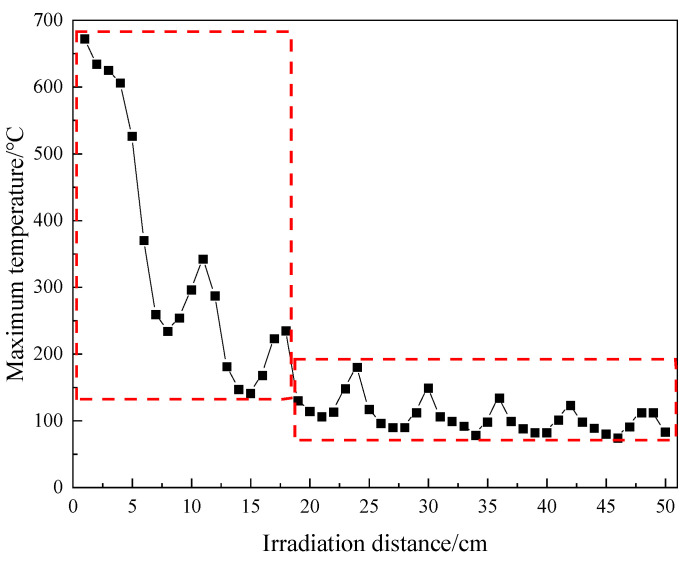
Relationship curve between the maximum temperature of the rock sample and irradiation distance.

The maximum temperature under a rectangular waveguide at different irradiation distances is shown in Figure 5. It is attenuated with the increase in transmission distance, and, as there are peaks and valleys, the temperature shows the trend of cosine decrease, with the distance between temperature local extremes being about a 1/4 wavelength. The red square in Figure 5 shows that the temperature rise curve fluctuates greatly under irradiation within a distance of 18 cm, and the temperature fluctuation is small at a distance greater than 18 cm.

The maximum temperature is higher than 600 °C under the irradiation within a distance of 4 cm. The thermal expansion and temperature gradient caused by this local high temperature are large enough to cause a serious rock fracture effect. Similarly, in the process of actual microwave irradiation on heterogeneous rocks, the temperature of microwave-sensitive minerals with high dielectric properties rises rapidly after microwave irradiation; the temperature gradient and thermal expansion between minerals are large, which will also lead to rock fracture [24]. The maximum temperature from 4 cm to 8 cm drops sharply from 606 °C to 234 °C, with a decrease of 372 °C. This is because, at a distance of 4 cm, the electromagnetic wave has not yet emanated to the upper and lower free boundaries of the air domain, but has concentrated on the surface of the rock sample. After a distance of 4 cm, the width of the air domain is large enough, and the electromagnetic wave diffuses into the air, loses part of its energy, and the temperature begins to drop sharply. The 8 cm irradiation distance is the minimum point of the first temperature peak, and the maximum temperature after this distance changes in a wave shape, but none of them exceed the maximum temperature of 370 °C obtained at the distance of 6 cm.

Microwave radiation has the best heating effect within a distance of 5 cm, but in the actual microwave-assisted excavation process, it may not be convenient to use a microwave antenna at such a short distance. It can also be seen from Figure 5 that the temperature obtained by a short irradiation distance can also be achieved by long irradiation distance. For example, the maximum temperature at 11 cm (342 °C) is close to that at 6 cm (370 °C), and the maximum temperature at 18 cm (235 °C) is close to that at 9 cm (254 °C).

**Figure 6:**
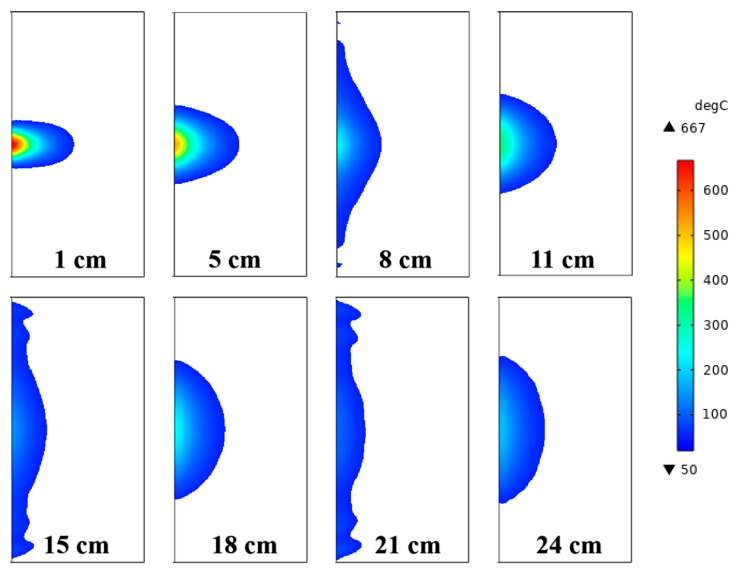
Temperature distribution in the rock sample under different irradiation distances.

The temperature distribution under different irradiation distances is shown in Figure 6. There are temperature extremes at the distances of 8 cm, 11 cm, 15 cm, 18 cm, 21 cm, and 24 cm. The area of the high-temperature zone of the maximum temperature is smaller than that of the minimum temperature point. With the increase in irradiation distance, the size of the high-temperature zone gradually changes from “narrow and deep” to “wide and shallow”. This is because the longer the distance, the larger the area of the rock surface covered by the electromagnetic wave due to its diffusion into the air, but the temperature gradually decreases. The long-distance microwave input energy can be increased in practical applications, to achieve a large-area weakening effect of the rock sample. In addition, the highest temperature of the rock is located on the rock surface, which is contrary to the conclusion that the center temperature is higher than the surface temperature obtained by the microwave cavity irradiation test and numerical simulation [25], and also indicates that the effect of open irradiation is different from that of cavity irradiation.

Teimori et al. [26] conducted radiation experiments on basalt with a microwave cavity and concluded that, at different radiation distances, the rock high-temperature zone presents the alternating distribution characteristics of a parallel microwave waveguide and vertical microwave waveguide. According to the 2D simulation results of the microwave surface irradiation at different irradiation distances, the high-temperature areas of the rock sample also appear alternately as “narrow and deep” and “wide and shallow”, which verifies the accuracy of the model presented in this paper.

The influence of different waveguide apertures was analyzed under irradiation at the distances of 5 cm, 8 cm, 11 cm, 15 cm, 18 cm, and 21 cm (5 cm, 11 cm, and 18 cm are short-, middle-, and long-wave crest distances, respectively; 8 cm, 15 cm, and 21 cm are short-, middle-, and long-wave trough distances, respectively).

### 3.3. Rock Sample Temperature under Different Waveguide Apertures

**Figure 7:**
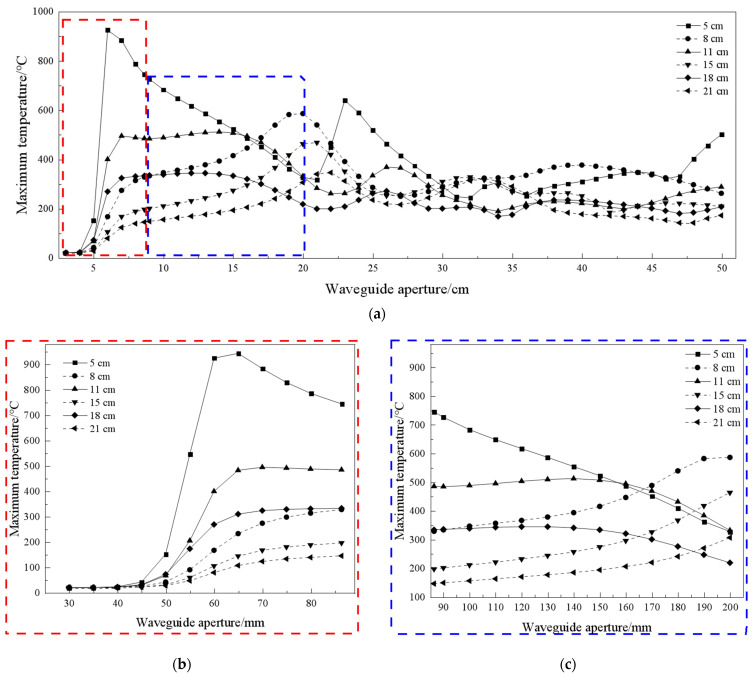
(**a**) Curves between the maximum temperature in the rock sample and the waveguide aperture. (**b**) Convergent waveguide (red dashed box); (**c**) horn waveguide (blue dashed box).

The curves of maximum temperature of the rock sample with waveguide apertures at different irradiation distances are shown in Figure 7. Waveguide aperture impacts the propagation of microwave energy, so the heating effect on the rock sample is different. It can be seen from Figure 7 that, if the waveguide aperture is greater than 40 mm, the rock sample begins to heat up. The electromagnetic wave can be transmitted from the waveguide aperture to the sample surface, which indicates that, in practical applications, the waveguide aperture cannot be very small, or it prevents the electromagnetic wave from being propagated to the sample surface.

The effect of different waveguide apertures on the temperature is also related to the location of the electromagnetic wave crest and valley in which the rock sample is located. Under the irradiation of the wave trough distance, the rock sample’s maximum temperature increases with the increase in waveguide aperture, within 200 mm. Under the irradiation of the wave crest distance, with the increase in waveguide aperture, the rock sample maximum temperature appears to first increase and stabilize to a certain value before decreasing.

The rock sample temperature with a waveguide aperture larger than 200 mm is lower than that within 200 mm. Under the irradiation of the wave trough distance, the rock maximum temperature appears under the irradiation of a 200 mm waveguide aperture, and the maximum temperature of the rock sample irradiated at other waveguide apertures is basically stable. Considering hob spacing, a value lower than 200 mm can be used as a suitable range of waveguide apertures arranged on the TBM cutterhead. With the increase in the horn waveguide aperture, the longitudinal propagation of the electromagnetic wave in the waveguide also appears as a crest and trough. Therefore, the maximum temperature of the rock samples is irregular with the waveguide aperture at different irradiation distances.

### 3.4. Damage in the Rock Sample

The change in waveguide aperture results in a different microwave energy transmission effect, so the temperature characteristic is quite different. However, the temperature characteristics of the rock cannot fully explain the microwave damage mechanism, and the damage characteristics of the rock sample are fundamental to evaluate the influence of the waveguide aperture. In this study, plastic strain was used to characterize the evolution of the microwave thermal damage and cracks in the rock. From the perspective of energy, the microwave damage evolution is the transformation of input microwave energy to the rock elastic strain energy and surface energy of cracks [27].

**Figure 8:**
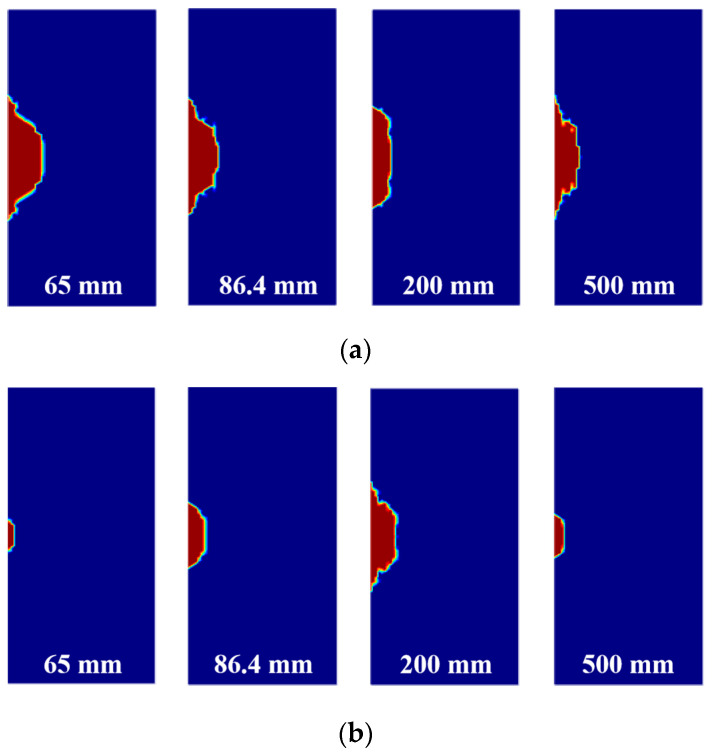
Distribution of the plastic zone and waveguide aperture under the irradiation time of 60 s. (**a**) Irradiation distance of 5 cm; (**b**) irradiation distance of 8 cm.

By comparing a 65 mm convergent waveguide, 86.4 mm standard waveguide, and 200 mm and 500 mm horn waveguides, the distribution of the plastic zone of the irradiated rock sample at the distances of 5 cm and 8 cm is shown in Figure 8. The red section represents the damaged zone of the sample. Consistent with the variation trend of the maximum temperature, under the irradiation time of 60 s, the convergence waveguide irradiation at the crest distance has a larger damage effect on the rock sample, while the trough distance has a smaller damage effect. When the irradiation distance is 5 cm, the convergent waveguide and the rectangular waveguide with a diameter of 65 mm have a large plastic zone. However, when the irradiation distance is 8 cm, the plastic zone of the 200 mm horn waveguide that irradiates the rock sample is large, and the plastic zone of the 500 mm waveguide that irradiates the rock sample is small; because of the further increase in the waveguide aperture, the electromagnetic wave is more divergent, and the rock temperature decreases, which leads to the decrease in plastic zone area.

**Figure 9:**
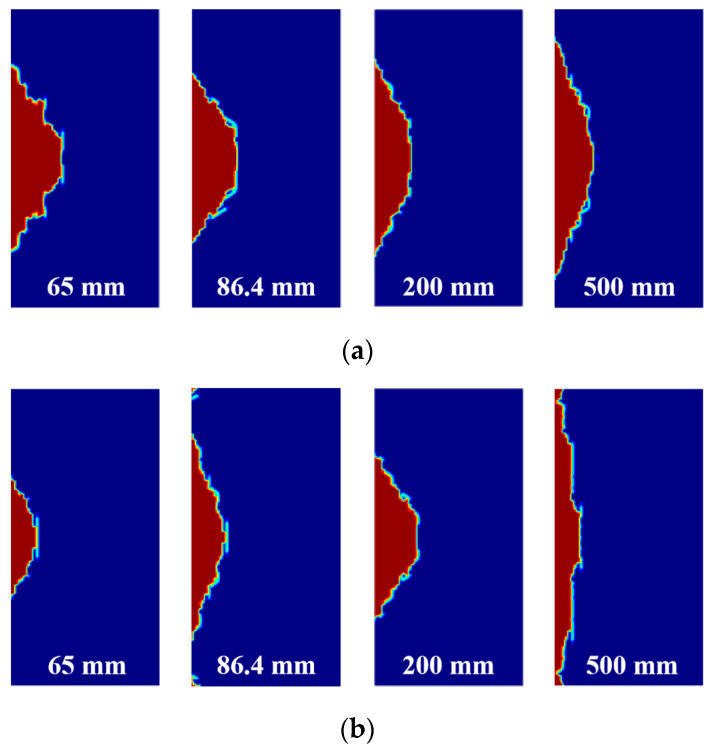
Plastic zone distribution of the rock sample and waveguide aperture under the irradiation time of 120 s. (**a**) Irradiation distance of 5 cm; (**b**) irradiation distance of 8 cm.

As shown in Figure 9, after increasing the exposure duration, that is, after increasing the input energy, the sample plastic zone generated by different waveguide apertures and irradiation distances increases significantly in both the width and depth directions. At the irradiation distance of 5 cm, the convergent waveguide irradiation applies great damage to the rock sample, while the horn waveguide irradiation has a wider damage coverage area. At the irradiation distance of 8 cm, the damage effect of the convergent waveguide on the rock is reduced, but the damage effect of the horn waveguide and rectangular waveguide is better. If a 500 mm waveguide aperture is used to irradiate rock, the damage can cover the entire rock sample surface.

At the wave crest distance, the plastic zone is larger. On the contrary, the plastic zone is smaller and wider at the trough distance. According to the analysis, the maximum temperature of the rock sample decreases with the increase in radiation distance and the waveguide aperture, but there is a larger high-temperature zone. By extending the irradiation time or increasing irradiation power, the overall weakening effect of microwave irradiation on the sample can be achieved.

## 4. Discussion

For an actual rock mass, the rock’s physical and mechanical parameters in different regions are quite different. Therefore, this paper qualitatively evaluated the temperature rise and damage characteristics at different waveguide distances and apertures by numerical simulation, providing ideas for a rational microwave layout in practical applications.

Great differences exist in the temperature rise and damage of the rock sample caused by open microwave irradiation and cavity irradiation. If a rock sample is placed in a cavity, it absorbs electromagnetic energy from all directions. Therefore, the rock temperature peak usually appears inside, the crack location and propagation direction are also uncertain, and the rock sample size is also limited by the cavity size. If the rock sample is irradiated in front of the open-ended waveguide, the rock sample is exposed to single-sided electromagnetic waves, and the high-temperature and damaged areas are located on the rock surface and within a certain thickness.

The electric field, temperature distribution, and damage characteristics in the rock sample under different irradiation distances show that the irradiation distance has a great influence on the weakening effect required for mechanical excavation. In mechanical excavation, the hard rock is peeled off between the hobs under the action of the cutting tool, and the large weakening area of the rock caused by microwave irradiation is the auxiliary effect required by the cutting tools on the soft rock. Short-distance microwave irradiation produces a high-temperature concentration area on the rock surface, and the weakening effect is “narrow but deep”, so it is suitable for hard rocks at a short distance of microwave irradiation. In the case of microwave irradiation at longer distance, the electromagnetic wave spreads all over rock surface, but the energy is weak. Increasing energy input can produce a larger area of rock weakening effect, so a rock with low intensity is suitable for microwave irradiation with a longer distance.

When microwave-assisted rock excavation is carried out, the irradiation distance is very small, which leads to a significant temperature rise. Operation procedures that take long processing times produce high-temperature side-effects on the cutterhead. The temperature should not reach the melting point of rocks. Therefore, controlling the appropriate irradiation distance not only achieves the same effect of weakening rocks by short-distance irradiation, but also ensures the safety of the TBM construction. Nekoovaght et al. [28] observed that the temperature rise of rocks varies nonlinearly with the irradiation distance, which corroborates our results. The simulation results show that the high-temperature zone at the wave crest distance is concentrated, and the high-temperature zone at the wave trough distance is wide. The wave crest and wave trough are affected by dielectric properties. In practical applications, it is necessary to pre-analyze the dielectric properties of the excavated rock and carry out the microwave irradiation test, to select the specific characteristic waveguide distance close to different rocks for construction.

**Figure 10:**
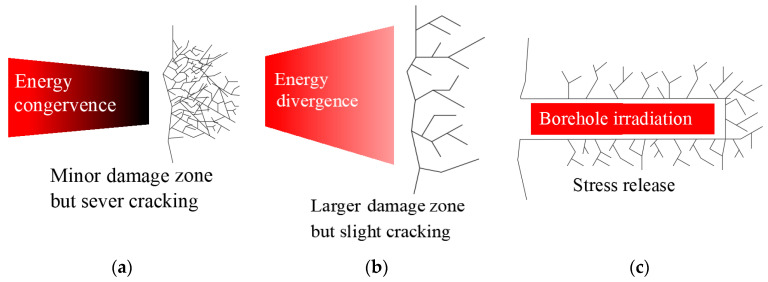
The effect of microwaves fracturing rocks. (**a**) Convergent waveguide irradiation; (**b**) horn waveguide irradiation; (**c**) borehole irradiation.

The heating and damage effects of diverse waveguide types and apertures are different. The convergent waveguide has a significant effect on the energy convergence of electromagnetic waves under the irradiation at a short crest distance, generating a certain range of high-energy areas, which has a good cracking effect on the local rock, as shown in Figure 10a. However, the increase in wave trough distance or irradiation distance leads to the decrease in energy in the high-energy region, and the rock is far away from the high-energy region. The radiation effect of the convergent waveguide gradually weakens. Under a short-distance irradiation, the effect of the horn waveguide is not as good as that of the convergent waveguide, but the horn waveguide has a larger damage width to the rock by energy divergence, as shown in Figure 10b.

In addition, the horn waveguide has a better radiation effect at a long distance or wave trough distance. If the waveguide aperture of the horn waveguide reaches a certain height and the power is increased appropriately, the rock surface will be sufficiently damaged. It is not favorable to achieve this effect under convergent waveguide irradiation. The use of a microwave-equipment-assisted cutting head drive has unique advantages, which can produce a large number of cracks in the rock within the irradiation range and reduce the excavation intensity of the TBM, but also help to release high ground stress energy and reduce the engineering safety risk caused by rock burst geological disasters. Lu et al. [29] proposed the method of borehole microwave irradiation, as shown in Figure 10, to prove that it is feasible to use microwave irradiation on rocks to alleviate a high in situ stress concentration, avoid rock burst geological disasters, and thus reduce the occurrence of underground engineering construction accidents.

## 5. Conclusions

Microwave-assisted rock breaking has great application prospects in underground engineering excavation, such as improving the efficiency of mechanical excavation and preventing rock burst disasters. Based on the arrangement of microwave antennas on the cutterhead in the actual mechanical tunneling, the influence and mechanism of waveguide aperture at different irradiated distances were studied. The main conclusions are as follows:

1. The maximum temperature of microwave open-surface irradiation is located on the rock surface, which is different from that of cavity irradiation, indicating that it is necessary to improve the theory of microwave-assisted rock breaking by using microwave open-surface irradiation;
2. The increase in irradiation distance leads to a cosine decreasing trend in rock temperature. Each temperature extreme point is about a 1/4 wavelength away from each other, and the temperature rise effect and rock damage at the wave crest distance are relatively concentrated, with the wave trough distance being relatively wide;
3. Within the range of 50~200 mm waveguide apertures, the damage of rocks irradiated within a 5 cm distance is better. When the irradiation condition within a 5 cm distance is not difficult to achieve, a 65 mm aperture convergent waveguide or rectangular waveguide can be selected for irradiation at the other crest distance, or a 200 mm aperture horn waveguide can be selected for irradiation at the trough distance, which has a better damage effect on the rock;
4. Convergent waveguides have a gathering effect on electromagnetic wave energy. The damage caused by short-wave crest irradiation to rocks is “narrow but deep”. The horn waveguide has a divergent effect on electromagnetic wave energy, making it spread over the rock surface with the increase in waveguide aperture. The damage to the rock sample is relatively “wide but shallow”. Therefore, the appropriate waveguide irradiation can be selected according to the actual demand of rock excavation.

## Figures and Tables

**Table 1:** Physical and mechanical parameters of rock.

Parameter	Value
Relative dielectric constant	8.6–1.35 j
Elastic modulus/GPa	90
Poisson’s ratio	0.22
Cohesion/MPa	30
Internal friction angle/rad	45
Thermal expansion coefficient/K^−1^	9.5 × 10^−6^
Thermal conductivity/W·m^−1^·K^−1^	2.9
Specific heat capacity/J·kg^−1^·K^−1^	850
Density/kg·m^−3^	2870

## Data Availability

The data presented in this study are not publicly available at this time, but may be obtained upon reasonable request from the authors.
